# Immunological and biochemical biomarker alterations among SARS-COV-2 patients with varying disease phenotypes in Uganda

**DOI:** 10.1186/s12879-023-08854-0

**Published:** 2023-12-06

**Authors:** Charles Drago Kato, Julius Nsubuga, Nixon Niyonzima, Annah Kitibwa, Enock Matovu, Emmanuel Othieno, Patrick Ssebugere, Amanda Agnes Tumwine, Monica Namayanja

**Affiliations:** 1 School of Bio-security, Biotechnical & Laboratory Sciences, College of Veterinary Medicine, Animal Resources & Bio-security, Makerere University, P.O Box 7062, Kampala, Uganda; 2 Uganda Cancer Institute, P.O Box 3935, Kampala, Uganda; 3 Department of Pathology, Soroti University, P.O. Box 211, Soroti, Uganda; 4 Department of Chemistry, College of Natural Sciences, Makerere University, P.O Box 7062, Kampala, Uganda

**Keywords:** COVID-19, SARS-CoV-2, Cytokine, Biomarkers, Inflammation

## Abstract

Every novel infection requires an assessment of the host response coupled with identification of unique biomarkers for predicting disease pathogenesis, treatment targets and diagnostic utility. Studies have exposed dysregulated inflammatory response induced by the novel severe acute respiratory syndrome coronavirus 2 (SARS-CoV-2) as significant predictor or cause of disease severity/prognosis and death. This study evaluated inflammatory biomarkers induced by SARS-CoV-2 in plasma of patients with varying disease phenotypes and healthy controls with prognostic or therapeutic potential. We stratified SARS-CoV-2 plasma samples based on disease status (asymptomatic, mild, severe, and healthy controls), as diagnosed by RT-PCR SARS-CoV-2. We used a solid phase sandwich and competitive Enzyme-Linked Immunosorbent Assay (ELISA) to measure levels of panels of immunological (IFN-γ, TNF-α, IL-6, and IL-10) and biochemical markers (Ferritin, Procalcitonin, C-Reactive Protein, Angiotensin II, Homocysteine, and D-dimer). Biomarker levels were compared across SARS-CoV-2 disease stratification. Plasma IFN-γ, TNF-α, IL-6, and IL-10 levels were significantly (*P* < 0.05) elevated in the severe SARS-CoV-2 patients as compared to mild, asymptomatic, and healthy controls. Ferritin, Homocysteine, and D-dimer plasma levels were significantly elevated in severe cases over asymptomatic and healthy controls. Plasma C-reactive protein and Angiotensin II levels were significantly (*P* < 0.05) higher in mild than severe cases and healthy controls. Plasma Procalcitonin levels were significantly higher in asymptomatic than in mild, severe cases and healthy controls. Our study demonstrates the role of host inflammatory biomarkers in modulating the pathogenesis of COVID-19. The study proposes a number of potential biomarkers that could be explored as SARS-CoV-2 treatment targets and possible prognostic predictors for a severe outcome. The comprehensive analysis of prognostic biomarkers may contribute to the evidence-based management of COVID-19 patients.

## Introduction

Coronavirus disease 2019 (COVID-19) is a global pandemic with great related deaths that started as an epidemic in Wuhan, China since 2019. It is caused by the novel severe acute respiratory syndrome coronavirus 2 (SARS-CoV-2) reported worldwide [1]. The disease primarily, affects the lungs with the virus entering the host cells through binding of the spike protein to the angiotensin-converting enzyme 2 receptor via the receptor binding domain of the S-protein [2]. Like other infections, patients infected with SARS-CoV-2 respond differently and might clinically manifest with fever, non-productive cough, dyspnoea, myalgia, fatigue, normal or decreased leukocyte counts, and radiographic evidence of pneumonia [3]. However, the majority of patients remain asymptomatic with or without detectable virus or experience mild upper air involvement while a small number progress to a severe potentially lethal disease the hallmark of which is an acute respiratory distress syndrome (ARDS) [4]. The ARDS is the main cause of death and is associated with pathological damage to the lungs and multiple organs within the body: heart, kidney, and liver, leading to multiple organ exhaustion [4, 5]. Although it is currently not yet clear why a portion of COVID-19 patients develop an ARDS, several studies have demonstrated that at this stage several inflammatory biomarkers are significantly increased.

The ARDS is characterized by cytokine storm with uncontrolled systemic inflammatory response resulting from release of pro-inflammatory cytokines and chemokines by immune cells [6]. This cytokine storm is associated with ferocious severe symptoms and multiple organ failure that eventually leads to death [3, 7–9]. Furthermore, hyperactivation of the inflammatory cascade leading to cytokine storm has been extensively cited as a critical biological response in patients with severe COVID-19 [10]. Studies have documented significant elevation of inflammatory cytokines TNF-α, IL-6 in severe COVID-19 patients compared to non-severe cases [3, 7–9, 11, 12]. Other inflammatory markers like C-reactive protein (CRP) have been shown to significantly increase from early stage with a positive correlation to disease severity [10–12]. A significant increase in total serum ferritin, CRP, Procalcitonin and D-dimer was registered in COVID-19 deaths compared to survivors [13–15]. A study in China, associated D-dimer levels of over 1 µg/mL with an increased risk of poor prognosis [17]. The viral load of COVID-19 patients detected from the respiratory tracts was positively associated with lung injury, disease severity and elevated plasma Angiotensin II level [11].

Until now, the factors surrounding this varied individual disease responses are not very clear. Preliminary studies investigating this response have pointed towards the cytokine storm and other inflammatory biomarkers as playing significant roles in predicting disease severity. Biomarker changes have been reported previously in COVID-19 cases but limited information about correlation with disease severity is known. This unpredictable disease course thus necessitates the immediate categorization of patients into risk groups after an initial diagnosis, to ensure optimal resource allocation and case management. We hypothesized that a number of biomarkers might be involved in the pathogenesis of SARS-CoV-2. The identification of effective biomarkers with potential to classify patients based on their risk is paramount to guarantee prompt treatment or identify patients with potential to suffer rapid disease progression to severe complications and death. Indeed, identified biomarkers unique to latent/asymptomatic cases could be harnessed for supportive therapy to manage severe cases or disease management at large.

## Materials and methods

### Study design and participants

We used a case-control study to analyze a panel of inflammatory and immunological markers using archived plasma and serum samples stored at the Integrated Biorepository of H3A Uganda (IBRH3AU) at Makerere University. Plasma and serum samples were collected from participants enrolled at Mulago National Referral Hospital, Entebbe Regional Referral Hospital, Jinja and Mbarara Regional Referral Hospitals during the first SARS-CoV-2 wave between January and July 2021. At the biorepository, specimen from both cases and controls were aliquoted and immediately stored at -80^o^C until further analysis. A “case” was defined as an individual with a positive nasopharyngeal swab SARS-CoV-2 test by reverse transcriptase-polymerase chain reaction (RT-PCR) [18] and a “control” as an individual with a negative SARS-CoV-2 test during voluntary testing or contact tracing. After disease confirmation, cases were categorised into asymptomatic, mild and severe following WHO clinical management criteria and Ministry of Health, Uganda [18, 19]. Asymptomatic cases were defined as those with no clinical symptoms, Mild cases were those with non-specific symptoms like Fever, fatigue, cough, sore throat, nasal congestion, headache, muscle pain or malaise and severe cases defined as those with severe pneumonia or acute respiratory distress syndrome [19–21]. All samples were analysed at the BSL2 Biomarker laboratory at the Centre for Biosecurity and Global Health, Makerere University.

Before storing specimen within the biobank, routine laboratory diagnosis of other infections including, malaria (Microscopic examination of wet and thick blood films from finger prick blood), helminths (microscopic examination of filtered urine for the presence of eggs), amoebiasis (detection of cysts in stool), typhoid (IgG/IgM), tuberculosis (sputum microscopy), Human Immunodeficiency Virus (rapid diagnostic test strip) and Urinary tract infections (urine test strip compared to colored scale) [23] were performed. For this study, positive SARS-CoV-2 samples, or controls with any of the above concurrent infection were not considered. In total, matched plasma and serum from 160 participants (40 asymptomatic, 40 mild, 50 severe and 30 healthy controls) were selected.

### Biochemical biomarker assays

Six biomarkers (Ferritin, Procalcitonin (PCT), C-Reactive Protein (CRP), Angiotensin II (Ang II), Homocysteine (HCY) and D-dimer) were assayed in plasma or serum using sandwich or competitive ELISA (MyBiosource, Inc, USA) as described by the manufacturer. Briefly, to the 96 well Microplate were added 50 µl standard solution and 40 µl test sample in triplicates followed by 10 µl anti-FTL antibody to sample wells, and 50 µl streptavidin-HRP to sample wells and standard wells. After mixing well, the plate was covered with sealer and incubated at 37 °C for 60 min. After 5 washes with at least 350 µl wash buffer for 1 min, added 50 µl substrate solution A and then 50 µl substrate solution B to each well. This was followed by incubation of plate covered with a new sealer for 10 min at 37 °C in the dark for colour development after which 50 µl Stop Solution was added to each well and the plate optical density values read using microplate reader at 450 nm (Biotek, UK).

### Immunological biomarker assays

Plasma concentrations of four cytokines (IFN-γ, TNF-α, IL-6, and IL-10) were assayed in triplicates using solid phase sandwich ELISA (BD OptEIA™, USA) as described previously [23, 24]. Briefly, microplates (nunc™, Denmark) were coated with 100 µl per well of capture antibody diluted in coating buffer (1x phosphate-buffered saline, PBS) and incubated over night at 4^o^C (Electrocool LG, South Korea). Microplates were aspirated and washed 3 times with 300 µl of wash buffer (1x PBS with 0.05% Tween-20). Microplates were then blocked with 200 µl per well of assay diluent (1x PBS with 10% fetal bovine serum albumin (Biochrom^AG^, German) and incubated for 1 h at room temperature (RT). After washing microplates 3 times with wash buffer, 200 µl assay diluent per well was added, followed by 100 µl plasma sample, 100 µl standards and incubated for 2 h at RT. After washing microplates 5 times as above, 100 µl per well working antibody detector (biotinylated detection antibody + Streptavidin-horseradish peroxidase) was added and incubated for 1 h at RT. After 7 washes, 100 µl per well substrate solution (tetramethylbenzidine, BD Biosciences, Belgium) was added and incubated in the dark for 30 min, after which 50 µl per well of stop solution (2M H_2_SO_4_) was added and the plate read at 450 nm using a microplate reader (Biotek, UK).

### Data analysis and management

All data was anonymized prior to analysis with numerical variables summarized using mean and standard deviation of mean. All comparisons of categorical variables cytokine and biochemical biomarker data were analyzed using Graphpad Prism 8.0 statistical packages. Comparison of categorical variables was performed using Chi-square test at significance level (*P* < 0.05, two-sided). Before statistical analysis of biomarker data, deviation from normality was tested using D’Agostino & Pearson omnibus normality test. Since data did not pass the normality test, all biomarker data was presented as medians. Comparison of biomarker data across asymptomatic, mild and severe cases was done using Kruskal-Wallis tests at a significant level (*P* < 0.05). Multiple comparisons across the different groups were done using Dunn’s multiple comparisons test at a significant level (*P* < 0.05). Correlation analysis between the different biomarkers were performed using bivariate non-parametric Spearman’s correlation rank test at a significant level (*P* < 0.05, two tailed).

## Results

### Baseline characteristics of participants

A total of 160 study participant matched plasma and serum (40 asymptomatic, 40 mild, 50 severe and 30 healthy controls) samples were retrieved from the biorepository. The ratio of male (112) to female (48) was approximately 2:1 with an average age of 40.6±15.2 years. Among the participants; healthy controls were 18.7% (30/160), 25.0% (40/160) were asymptomatic, 25.0% (40/160) were mild, while 31.3% (50/160) were severe cases (Table 1).

**Table 1:** Baseline characteristics of participants

Characteristics	Participant status				
	Controls	Asymptomatic	Mild	Severe	Total n (%)
Subjects n (%)	30(18.7)	40(25.0)	40(25.0)	50(31.3)	160 (100.0)
Average age	36.5 ± 14.5	36.0 ± 11.5	34.6 ± 10.5	51.4 ± 16.1	40.6 ± 15.2
Age group (years)					
<18 18–35 36–49 ≥50	1 14 10 5	1 18 15 6	2 21 14 3	0 9 16 25	4(2.5) 62(38.7) 55(34.4) 39(24.4)
Sex					
Male Female	14 16	36 4	32 8	30 20	112(70.0) 48(30.0)

### Biochemical biomarker levels and disease progression

**Fig. 1:**
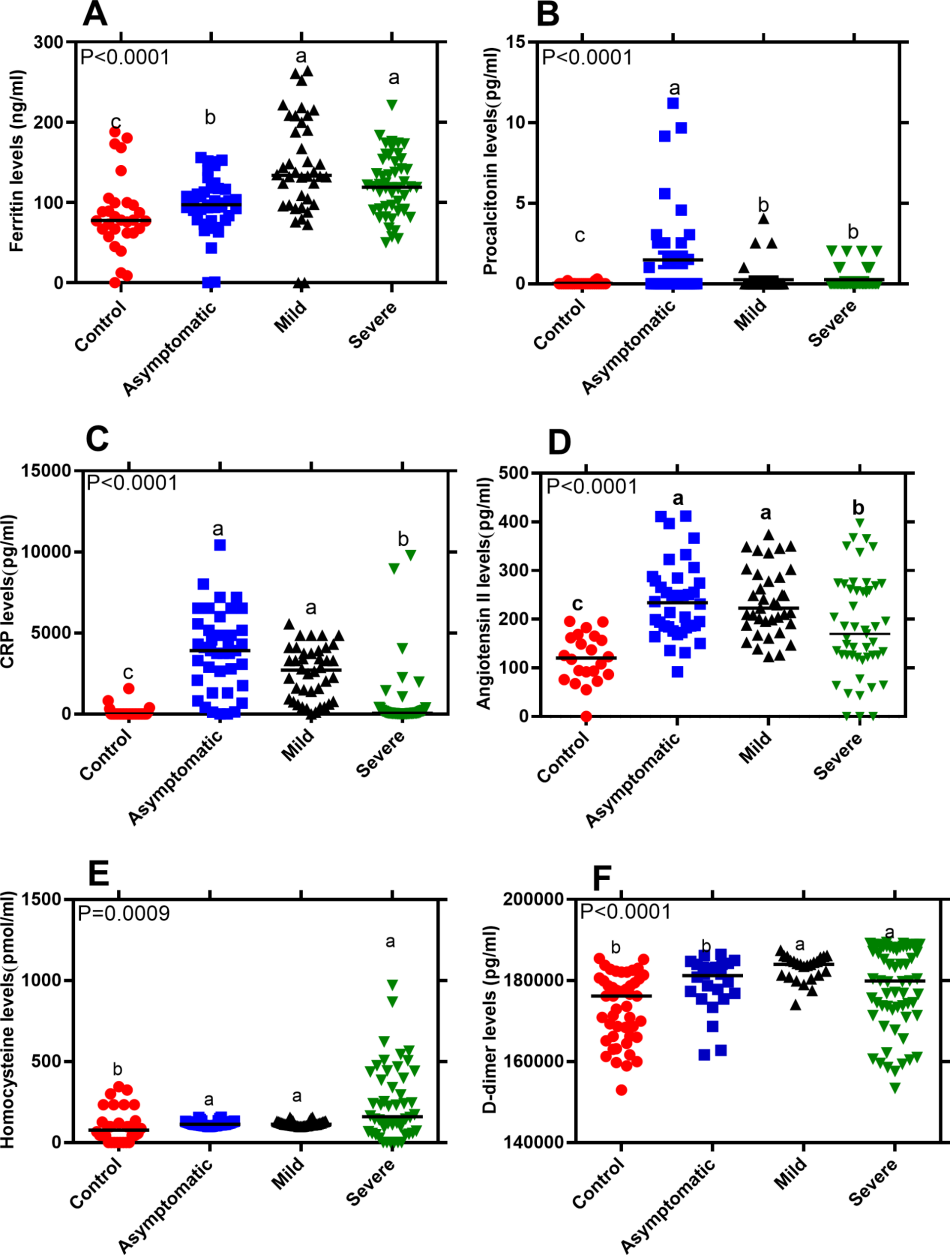
Plasma biochemical biomarker levels. Healthy controls (n = 30), asymptomatic (n = 40), mild (n = 40) and severe (n = 50). Scatter plot with horizontal line indicating biomarker median levels. Each dot defines an individual. Lower case letters (a > b > c) indicate level of significance between participant groups (Nonparametric Kruskal-Wallis, Dunn’s multiple comparisons tests, *P* < 0.05)

The detection limits for biomarker assays of Ferritin, Homocysteine, Procalcitonin, C-Reactive Protein, Angiotensin II and D-dimer were 0.31ng/ml, 4.7 pmol/ml, 3.9 pg/ml, 23.3 pg/ml, 18.7 pg/ml, and 255.3pg/ml respectively, calculated according to Armbruster and Pry [26]. Our data showed that median plasma levels of Ferritin, PCT, CRP, Ang II, HCY and D-dimer differed significantly among the study groups (Fig. 1A and B C, 1D, 1E, and 1 F; One-way ANOVA, *P* < 0.05). When median biomarker levels were compared across study groups, Ferritin, HCY and D-dimer (*P* = 0.04) plasma levels were significantly elevated in severe cases over asymptomatic and healthy controls. No significant differences in Ferritin, HCY, and D-dimer levels were observed between severe and mild cases, although median biomarker levels remained significantly higher in mild cases compared to asymptomatic cases and health controls. Similarly, there was significant elevation of ferritin and HCY levels in asymptomatic cases over healthy controls.

Plasma CRP and Ang II median levels were significantly higher in mild and asymptomatic cases as compared to severe cases and healthy controls. However, for both CRP and Ang II no significant differences were observed between mild and asymptomatic cases. Median plasma PCT levels remained significantly higher in asymptomatic cases when compared with other groups. No differences in PCT levels were observed between mild and severe cases, although these remained elevated over healthy controls.

### Immunological markers and disease progression

**Fig. 2A:**
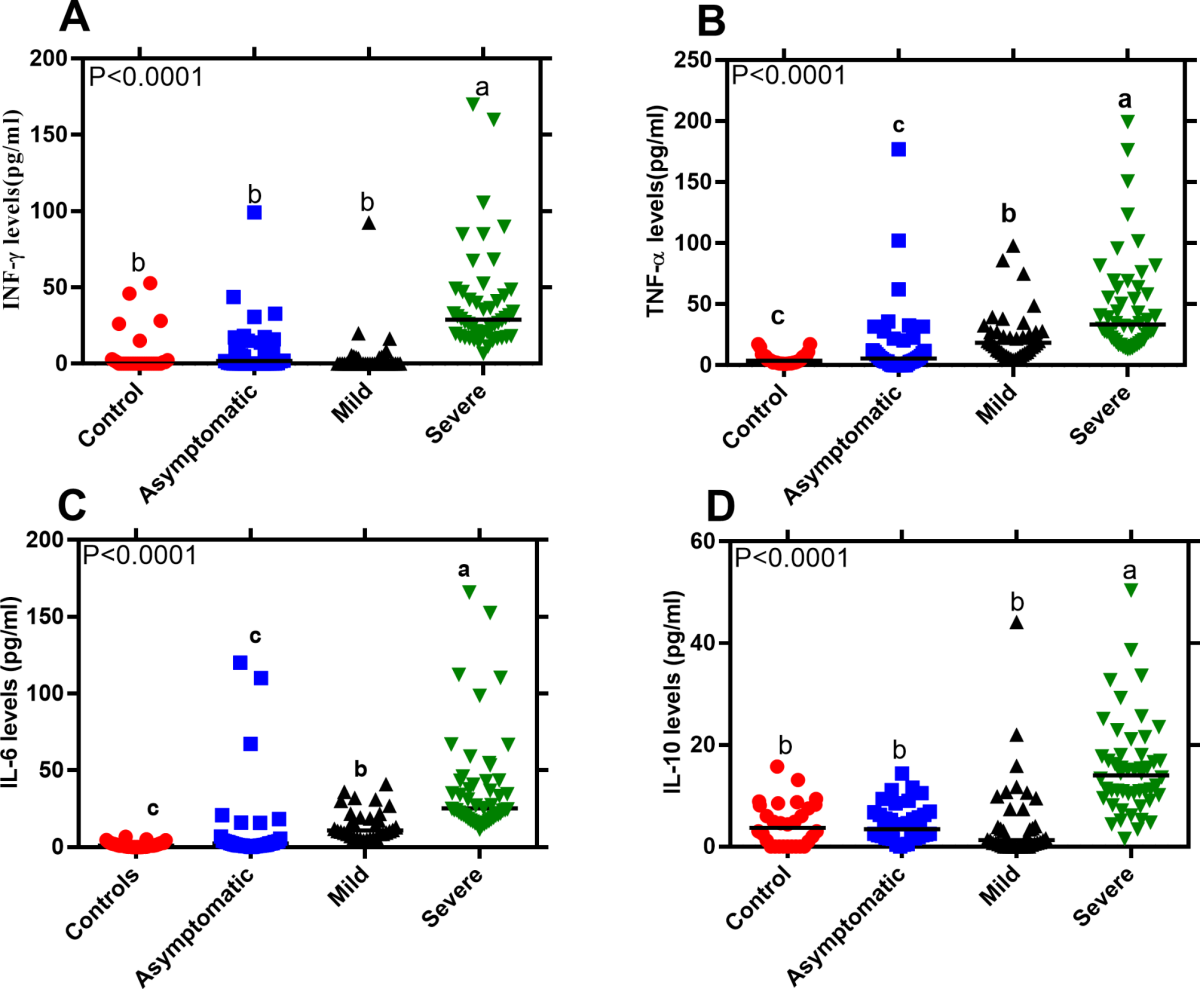
(**A**-**D**) Plasma cytokine levels of SARS-COV-2 patients and healthy controls. Participants of healthy controls (n = 30), asymptomatic (n = 40), mild (n = 40) and severe (n = 50) were involved. Scatter plot with horizontal line indicating biomarker median levels. Each dots defines an individual. Lower case letters (a > b > c) indicate level of significance between participant groups (Nonparametric Kruskal-Wallis, Dunn’s multiple comparisons tests, *P* < 0.05)

The detection limits for cytokine assays for IFN-γ, TNF-α, IL-6 and IL-10 were 8.3, 9.1, 3.6, 4.2pg/ml respectively, calculated according to Armbruster and Pry [26]. The results showed that median plasma levels (pg/ml) of IFN-γ, TNF-α, IL-6 and IL-10 differed significantly among study groups (Fig. 2A-D; Kruskal-Wallis; Dunn’s multiple comparisons test, *P* < 0.05). Plasma IFN-γ, TNF-α, IL-6 and IL-10 levels were significantly (*P* < 0.05) elevated in severe cases as compared to mild, asymptomatic, and healthy controls. Plasma cytokine levels were significantly higher in mild than asymptomatic cases and healthy controls for only TNF-α and IL-6. When median cytokine levels were compared between asymptomatic individuals and healthy controls, no significant statistical differences were noted for the four cytokine markers, IFN-γ (*P* > 0.99), TNF-α (*P* = 0.54), IL-6 (*P* = 0.13) and IL-10 (*P* > 0.99).

### Correlation between biomarkers levels in SARS-COV2 patients

Spearman’s correlation rank test was performed to investigate the association between biomarker plasma levels for mild and severe cases. For mild cases, a significant positive correlation was observed between IFN-γ with IL-10 (Spearman r = 0.54, *P* = 0.0004, Spearman’s rank correlations *p* < 0.05, two tailed; Table 2). Significant negative correlations were observed between IL-6 with Ang II (Spearman r=-0.60, *P* = 0.0003), Ferritin with Ang II (Spearman r=-0.46, *P* = 0.004) among COVID-19 mild cases.

**Table 2:** Spearman Correlation coefficient between biomarker levels among for mild cases

Cytokine	Cytokine and Biomarker correlation coefficient r_s_									
	IFN-γ	TNF-α	IL-6	IL-10	Ferritin	PCT	CRP	Ang II	HCY	D-dimer
IFN-γ		0.141	0.029	0.541***	0.235	-0.177	-0.083	-0.083	-0.238	-0.322
TNF-α			0.002	0.258	-0.106	-0.233	0.186	0.419	0.159	-0.125
IL-6				-0.131	0.252	-0.316	0.209	-0.605***	0.015	-0.184
IL-10					-0.028	-0.105	0.082	0.084	0.001	-0.047
Ferritin						-0.040	0.010	-0.463**	-0.216	0.355
PCT							-0.028	-0.166	0.045	0.238
CRP								-0.273	0.101	0.196
Ang II									0.194	-0.295
HCY										-0.141

Spearman’s (r_s_) rank correlations were computed and statistical significance was considered at (*p* < 0.05*, *p* < 0.005**, *p* < 0.001*** and *p* < 0.0001****), Negative (-) = denotes negative correlation

For severe cases, significant positive correlations were observed between IFN-γ with TNF-α (Spearman r = 0.37, *P* = < 0.0001), IFN-γ with IL-10 (Spearman r = 0.48, *P* = < 0.0001), IFN-γ with Ang II (Spearman r = 0.32, *P* = 0.028), IFN-γ with HCY (Spearman r = 0.36, *P* = 0.012), TNF-α with Ang II (Spearman r = 0.32, *P* = 0.027), TNF-α with HCY (Spearman r = 0.35, *P* = 0.011), IL-6 with HCY (Spearman r = 0.31, *P* = 0.02), CRP with Ang II (Spearman r = 0.45, *P* = 0.0011, Spearman’s rank correlations *p* < 0.05, two tailed; Table 3).

**Table 3:** Correlation coefficient between biomarker levels for Severe cases

Cytokine	Cytokine and Biomarker correlation coefficient r_s_									
	IFN-γ	TNF-α	IL-6	IL-10	Ferritin	PCT	CRP	Ang II	HCY	D-dimer
IFN-γ		0.368****	-0.357****	0.479****	0.199	-0.089	0.195	0.319*	0.359*	0.144
TNF-α			0.126	-0.050	-0.004	-0.217	0.102	0.316*	0.354*	0.262
IL-6				0.256	0.155	-0.212	-0.301*	-0.148	0.319*	0.166
IL-10					0.125	-0.064	0.005	0.167	0.183	0.047
Ferritin						-0.202	0.010	0.001	0.032	0.082
PCT							-0.034	-0.132	0.004	-0.140
CRP								0.451**	-0.150	-0.053
Ang II									0.279	0.207
HCY										0.153

Spearman’s (r_s_) rank correlations were computed and statistical significance was considered at (*p* < 0.05*, *p* < 0.005**, *p* < 0.001*** and *p* < 0.0001****), Negative (-) = denotes negative correlation

## Discussion

Clinical management of SARS-COV2 in resource-limiting countries has always been a challenge [27]. In low-to-middle-income countries, the capacity of healthcare systems is constrained as observed during the COVID-19 outbreak, leading to worse clinical outcomes. In such scenarios, early disease diagnosis and stratification of patients according to severity would facilitate the allocation of the limited medical resources. To this effect, the identification of novel biomarkers that are effective in clinical management would be helpful [28]. In the current study, we analysed a profile of biomarkers in light of the varying COVID-19 disease phenotypes.

Our data shows that plasma concentrations IFN-γ, TNF-α, IL-6, IL-10, Ferritin, HCY, and D-dimer levels were significantly elevated in severe cases as compared to mild, asymptomatic, and healthy controls. The up-regulated inflammatory biomarkers may lead to abnormal systemic inflammatory responses that cause disease severity and have been proposed to be predictive of disease severity and poor prognosis [14]. A significant increase in total serum Ferritin, PCT, CRP, and D-dimer was registered in COVID-19 deaths as compared to survivors [10, 13–15]. Studies in China, patients with D-dimer levels over 1 µg/ml showed an increased risk of poor prognosis [17] and ≥ 2 µg/ml predicted mortality to death [16]. Elevated D-dimer was observed in severe cases over mild conditions thus the prominent blood coagulation changes in SARS-CoV-2 infection [28, 29]. Higher D-dimer was significantly associated with severity and mortality of disease among critical cases induced by SARS-CoV-2 [31]. Elevated Ferritin, a storage molecule for iron metabolism has been proposed to be directly involved in COVID-19 and might be a predictive biomarker for disease progression [32]. However, in another Italian study, Ferritin was associated with severe lung involvement albeit with no worse prognosis [33]. In other COVID-19 studies, an upregulation in Ferritin and CRP in COVID-19 cases was associated with poor clinical prognosis [34]. C-reactive protein (CRP) is secreted by the liver in response to inflammation and inflammatory cytokines due to infection. Similar to our current study, CRP has been shown to significantly increase from early stage with a positive correlation to disease severity [10–12, 34, 35, 39]. Higher D-Dimer and CRP were significantly associated with mortality and severity of disease among critical cases induced by SARS-CoV-2 [33, 40, 41]. Indeed, CRP has been demonstrated to have good diagnostic accuracy in the early prediction of severe COVID-19 patients with sensitivity and specificity of 83% and 91% respectively [39].

Homocysteine is involved in SARS-CoV-2 virus metabolism and Angiotensin II receptor activation and has been reported to be associated with severe disease [37, 38]. In this study, although Angiotensin II levels were significantly elevated in cases over controls, mild cases demonstrated higher levels compared to severe cases. In other studies, Angiotensin II has been associated with severe COVID-19 [39, 40]. In a study where Ang II levels were significantly elevated was linearly correlated with viral load and lung injury in COVID-19 cases thus predictive of disease severity [11]. Angiotensin II stimulates/induces the expression of a multifunctional IL-6 thus contributes to cytokine storm with poor outcome/prognosis in COVID-19 patients [41, 42]. Angiotensin II is a central effector molecule of activated Renin-Angiotensin system and elevated levels have been associated with severe COVID-19 [46]. Plasma Procalcitonin levels were significantly higher in asymptomatic than in mild, severe cases and healthy controls. This is contrary to the previous study that demonstrate high procalcitonin levels to be associated with severe COVID-19 infections in patients and proposed as a prognostic biomarker [47]. Patients with viral infections demonstrated no elevation of Procalcitonin serum levels unlike elevation of serum procalcitonin in patients with bacterial infection [48]. However, procalcitonin is considered not to be a reliable prognostic biomarkers in several infections [49].

The up-regulated cytokines (IFN-γ, TNF-α, IL-6, IL-10) in our study indicate induced activation of immune responses against SARS-COV-2 infection and are consistent with other COVID-19 studies [36]. Pro-inflammatory cytokine (IFN-γ, TNF-α, IL-6) are activated for the effective nonspecific antiviral infections by inducing immune cells through activation of intracellular signalling pathways between infected and uninfected cells. This in turn recruits lymphocytes and leukocytes to the infection site [50]. The elevated pro-inflammatory cytokines levels may lead to abnormal systemic immune inflammatory responses that cause disease severity. Several studies have documented significant elevation in inflammatory cytokines (IFN-γ, TNF-α, IL-6) in severe COVID-19 compared to non-severe cases [3, 7–9] that are consistent with this study and may be proposed as prognostic markers. Higher levels of IL-6 and TNF-α were associated with disease severity in COVID-19 patients and significant predictors of mortality and survival [45, 46]. Pleiotropic cytokines IL-6 and IL-10 expression may predict early diagnosis of disease severity [35, 47] and IL-6 has been associated with mortality risks [53]. These should be considered in the predictive disease prognosis, treatment and management of COVID-19 patients. Nevertheless, mediation to decrease inflammation will negatively upset viral clearance. However, dysregulated excessive pro-inflammatory cytokines might lead to disease pathogenicity, severity and mortality if not regulated by anti-inflammatory cytokines. Therefore, this explains why patients with severe SARS-COV2 in our study exhibited elevated IL-10 plasma level.

Even though the inflammatory markers reported in our study have been reported elsewhere in SARS-COV2 infections, our findings provide immunological information that could be used for the development of vaccines and drugs for other respiratory-based infections and future cases of flu-like outbreaks. A similar approach was used to get a deeper understanding of immunological responses in SARS-COV2 when compared with earlier outbreaks of SARS and MERS [54–56].

Our work has some limitations that need to be considered when interpreting our observed results. We used biobank specimens collected for other requirements and as such, the presence of other comorbidities like hypertension and diabetes was not ascertained yet these have been shown to affect SARS-COV2 disease severity [57]. Due to a small sample size when categorized by age, sex, and ethnicity, we could not adjust for these predictors that have been shown to affect SARS-COV-2 disease severity [58, 59].

## Conclusion and recommendations

In summary we report variations in inflammatory biomarker levels across SARS-COV-2 patient stratifications. Given the limited access and cost associated with Intensive Care Units (ICU) in resource limiting countries, these biomarkers could be explored as predictors for severe outcome that could require ICU admission. We also demonstrate that certain biomarkers are associated with asymptomatic cases and these could be explored as therapeutic targets to modulate disease progression. Similarly, cytokine markers (IFN-γ, TNF-α, IL6 and IL10), were associated with severe patients and these could be explored as therapeutic targets. Therefore, studies exploring the prognostic and therapeutic role of these identified biomarkers will be helpful in the management of COVID-19 patients.

## Data Availability

All the data used to support the findings of this study are included within the article.

## Abbreviations

SARS: CoV-2 Severe acute respiratory syndrome coronavirus 2
COVID: 19 Coronavirus Disease 2019
ELISA Enzyme: Linked Immunosorbent Assay
IL: 6 Interleukin-6
IL: 10 Interleukin-10
TNF: αTumor necrosis factors-α
IFN: γInterferon gamma
ARDS: Acute respiratory distress syndrome
CRP: C-reactive protein
RT-PCR: Reverse transcriptase-polymerase chain reaction
BSL: Biosafety Level
WHO: World Health Organization
PCT: Procalcitonin
HCY: Homocysteine
HRP: Horseradish peroxidase
RT: Room temperature
